# Supplementary material for: Transcriptional profiling of lung macrophages identifies a predictive signature for inflammatory lung disease in preterm infants
Source: Commun Biol. 2020 May 22;3:259. doi: 10.1038/s42003-020-0985-2 (PMC7244484; doi:10.1038/s42003-020-0985-2)
Supplement: Supplementary file 1 — Supplementary Information [file 42003_2020_985_MOESM1_ESM.pdf]

**Transcriptional Profiling of Lung Macrophages Identifies a Predictive Signature for Inflammatory Lung Disease in Preterm Infants**

**Authors:** Debashis Sahoo, Livia S. Zaramela, Gilberto E. Hernandez, Uyen Mai, Sahar Taheri, Dharanidhar Dang, Ashley N. Stouch, Rachel M. Medal, Alyssa M. McCoy, Judy L. Aschner, Timothy S. Blackwell, Karsten Zengler, Lawrence S. Prince

**Supplementary Figures**

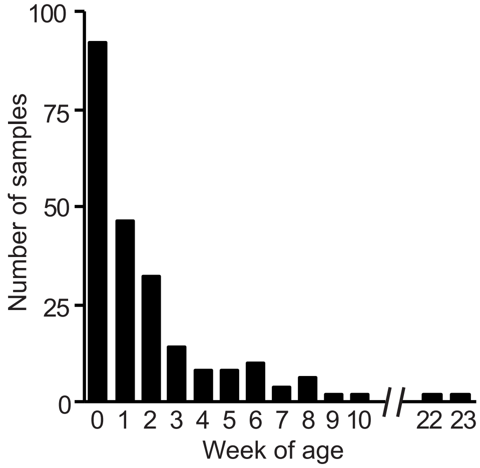


**Supplementary Figure 1.** Sample distribution by chronological age. Not every patient was analyzed at each time point. Reasons for missing samples included extubation and less invasive respiratory support and lack of sufficient cell number in the tracheal aspirate. In addition, some patients were intubated at later time points, making them eligible for analysis. For example, we obtained data from 42 patients on day 1. We also obtained day 7 samples from 12 of the 42 patients that also had day 1 samples. In addition, we obtained data on day 7 from an additional 11 patients that did not have data on day 1, giving a total of 23 patient samples on day 7. The sample number in the graph above includes both control and LPS-treated samples.

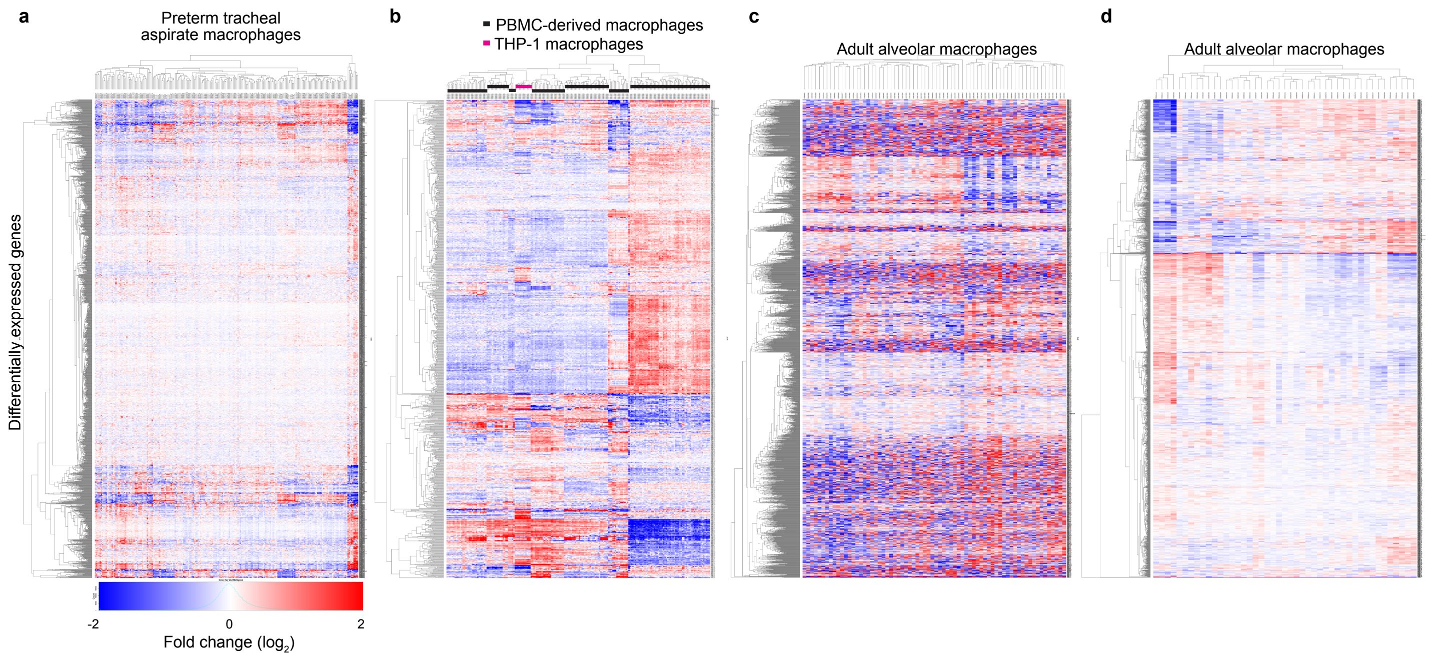


**Supplementary Figure 2.** Global gene expression in tracheal aspirate macrophages from preterm infants. Unsupervised hierarchical clustering analysis of all tracheal aspirate macrophage samples (**a**) compared to published datasets from human peripheral blood monocyte-derived macrophages (PBMC), the human THP-1 macrophage cell line (**b**), and two different studies examining adult alveolar macrophages (**c,d**). Global patterns of expression demonstrate the unique gene expression profile of lung macrophages from human preterm infants compared to other macrophage datasets.

**Supplementary Table**

| **Characteristic** | **n** | **%** |
|---|---|---|
| total | 128 | 100% |
| female | 51 | 39.8% |
| mean gestational age (wk) | 26 2/7 | |
| median gestational age (wk) | 26 6/7 | |
| mean birth weight (g) | 871 | |
| chorioamnionitis | 28 | 21.9% |
| vaginal delivery | 31 | 24.2% |
| survival | 118 | 92.2% |
| mean days on ventilator | 18.3 | |
| BPD | 84 | 73.7% |
| none | 30 | 26.3% |
| mild | 9 | 7.9% |
| moderate | 28 | 24.6% |
| severe | 47 | 41.2% |

**Supplementary Table**. Characteristics of patients enrolled in the study. BPD outcome and severity categorized using National Institutes of Health criteria.

**Supplementary Methods**

| Target | Forward | Reverse |
|---|---|---|
| GAPDH | CTC ATG ACC ACA GTC CAT GC | CAC ATT GGG GGT AGG AAC AC |
| CCL3 | AGT TCT CTG CAT CAC TTG CTG | CGG CTT CGC TTG GTT AGG AA |
| CCL20 | TGC TGT ACC AAG AGT TTG CTC | CGC ACA CAG ACA ACT TTT TCT TT |

Oligonucleotide sequences used for Real Time PCR.
