# Supplementary material for: Transcriptional profiling of lung macrophages identifies a predictive signature for inflammatory lung disease in preterm infants
Source: Commun Biol. 2020 May 22;3:259. doi: 10.1038/s42003-020-0985-2 (PMC7244484; doi:10.1038/s42003-020-0985-2)
Supplement: Supplementary file 2 — Reporting Summary [file 42003_2020_985_MOESM2_ESM.pdf]

nature research

Corresponding author(s): Lawrence S. Prince

Last updated by author(s): April 23, 2020

Reporting Summary

Nature Research wishes to improve the reproducibility of the work that we publish. This form provides structure for consistency and transparency in reporting. For further information on Nature Research policies, see Authors & Referees and the Editorial Policy Checklist.

Statistics

For all statistical analyses, confirm that the following items are present in the figure legend, table legend, main text, or Methods section.

n/a	Confirmed	
☐	☒	The exact sample size (*n*) for each experimental group/condition, given as a discrete number and unit of measurement
☐	☒	A statement on whether measurements were taken from distinct samples or whether the same sample was measured repeatedly
☐	☒	The statistical test(s) used AND whether they are one- or two-sided<br>*Only common tests should be described solely by name; describe more complex techniques in the Methods section.*
☐	☒	A description of all covariates tested
☐	☒	A description of any assumptions or corrections, such as tests of normality and adjustment for multiple comparisons
☐	☒	A full description of the statistical parameters including central tendency (e.g. means) or other basic estimates (e.g. regression coefficient) AND variation (e.g. standard deviation) or associated estimates of uncertainty (e.g. confidence intervals)
☐	☒	For null hypothesis testing, the test statistic (e.g. F, t, r) with confidence intervals, effect sizes, degrees of freedom and P value noted<br>*Give P values as exact values whenever suitable.*
☒	☐	For Bayesian analysis, information on the choice of priors and Markov chain Monte Carlo settings
☒	☐	For hierarchical and complex designs, identification of the appropriate level for tests and full reporting of outcomes
☒	☐	Estimates of effect sizes (e.g. Cohen's d, Pearson's r), indicating how they were calculated

Our web collection on statistics for biologists contains articles on many of the points above.

Software and code

Policy information about availability of computer code

Data collection	No proprietary software was need to obtain publicly available data from GEO.
Data analysis	Details on software programs, versions, and sources are included in the manuscript text.

For manuscripts utilizing custom algorithms or software that are central to the research but not yet described in published literature, software must be made available to editors/reviewers. We strongly encourage code deposition in a community repository (e.g. GitHub). See the Nature Research guidelines for submitting code & software for further information.

Data

Policy information about availability of data

All manuscripts must include a data availability statement. This statement should provide the following information, where applicable:

- Accession codes, unique identifiers, or web links for publicly available datasets
- A list of figures that have associated raw data
- A description of any restrictions on data availability

Our data have been submitted through the GEO and will be made publicly available upon publication of the manuscript.

Field-specific reporting

Please select the one below that is the best fit for your research. If you are not sure, read the appropriate sections before making your selection.

☒ Life sciences ☐ Behavioural & social sciences ☐ Ecological, evolutionary & environmental sciences

For a reference copy of the document with all sections, see nature.com/documents/nr-reporting-summary-flat.pdf

Life sciences study design

All studies must disclose on these points even when the disclosure is negative.

Sample size	This study was a first of its kind. As a result, we had no previous information regarding standard deviation or effect of covariates on gene expression levels. We approximated needing around 100 patients for the study, knowing that some samples would not yield sufficient numbers of cells and therefore mRNA. We were able to obtain samples from 113 patients.
Data exclusions	No data were excluded from our study.
Replication	Replicate measures were only performed for real time PCR measurements in Figure 1d.
Randomization	Patients and samples were not assigned to different experimental groups up front.
Blinding	While blinding was not required for this study, data analysts were not aware of disease-related covariates at the time of initial analysis. Disease groups were assigned numerical values to mask the clinical variables.

Reporting for specific materials, systems and methods

We require information from authors about some types of materials, experimental systems and methods used in many studies. Here, indicate whether each material, system or method listed is relevant to your study. If you are not sure if a list item applies to your research, read the appropriate section before selecting a response.

Materials & experimental systems

n/a	Involved in the study	
☒	☐	Antibodies
☒	☐	Eukaryotic cell lines
☒	☐	Palaeontology
☒	☐	Animals and other organisms
☐	☒	Human research participants
☐	☒	Clinical data

Methods

n/a	Involved in the study	
☒	☐	ChIP-seq
☒	☐	Flow cytometry
☒	☐	MRI-based neuroimaging

Human research participants

Policy information about studies involving human research participants

Population characteristics	the following clinical information was obtained on each patient: BPD severity, birth weight, gender, gestational age at birth, weight at 36 wk corrected age, diagnosis of pneumonia, respiratory support at 36 wk, hospital days, days of mechanical ventilation, CPAP, nasal cannula oxygen, maternal chorioamnionitis, maternal hypertension, maternal pre-eclampsia, pulmonary hemorrhage, timing of initial intubation.
Recruitment	Study participants were premature infants less than 30 weeks gestation, who were intubated for mechanical ventilation due to respiratory distress syndrome. Samples were collected from participants weekly as long as they remained intubated. Patients were recruited from the neonatal intensive care units at Vanderbilt University, Rady Children's Hospital, and the University of California, San Diego. Some of the participating infants at Vanderbilt were enrolled in the Prematurity and Respiratory Outcome Program (PROP) at Vanderbilt University Medical Center (NCT01460576).
Ethics oversight	This study was approved by the Institutional Review Boards at Vanderbilt University and the University of California, San Diego. Of note, Rady Children's Hospital and the University of California, San Diego use a shared IRB structure.

Note that full information on the approval of the study protocol must also be provided in the manuscript.

Clinical data

Policy information about clinical studies

All manuscripts should comply with the ICMJE guidelines for publication of clinical research and a completed CONSORT checklist must be included with all submissions.

Clinical trial registration	N/A. While this study did use samples obtained from human subjects, the tracheal aspirates were obtained as part of routine clinical care and would have been otherwise discarded. No clinical interventions were included in our study and the medical providers were not notified of subject participation. Informed consent to use the samples and obtain clinical data as above was obtained from each patient's mother.
Study protocol	Study protocols were reviewed and approved by the Institutional Review Boards at Vanderbilt University and the University of California, San Diego. Of note, Rady Children's Hospital and the University of California, San Diego use a shared IRB structure.

Data collection	De-identified clinical data and demographic data approved by the IRB were obtained from the electronic medical record and stored in a secured, encrypted database.
Outcomes	For this non-interventional study, clinical outcome data approved by the IRB were obtained from the electronic medical record and stored in a secured, encrypted database.
