# Supplementary material for: Transcriptional profiling of lung macrophages identifies a predictive signature for inflammatory lung disease in preterm infants
Source: Commun Biol. 2020 May 22;3:259. doi: 10.1038/s42003-020-0985-2 (PMC7244484; doi:10.1038/s42003-020-0985-2)
Supplement: Supplementary file 3 — Description of Additional Supplementary Files [file 42003_2020_985_MOESM3_ESM.pdf]

Description of Additional Supplementary Files

Supplementary Data 1. List of all patient samples included in this study.

Supplementary Data 2. Differentially expressed genes in Figure 1.

Supplementary Data 3. Reactome categories for differentially expressed genes on days 1 and 7.

Supplementary Data 4. Differentially expressed genes following LPS treatment in Figure 2.

Supplementary Data 5. List of innate immune genes in Figure 3.

Supplementary Data 6. Genes identified by StepMiner in Figure 5.

Supplementary Data 7. Reactome categories for genes with time-dependent increases in expression as identified by StepMiner in Figure 5f.

Supplementary Data 8. Source data for Figure 1d
